# Myricetin enhance chemosensitivity of 5-fluorouracil on esophageal carcinoma in vitro and in vivo

**DOI:** 10.1186/s12935-014-0071-2

**Published:** 2014-07-18

**Authors:** Lei Wang, Jianfang Feng, Xiaonan Chen, Wei Guo, Yuwen Du, Yuanyuan Wang, Wenqiao Zang, Shijie Zhang, Guoqiang Zhao

**Affiliations:** 1 Department of Emergency, The First Affiliated Hospital of Zhengzhou University, No.1, Jianshe Road, Zhengzhou 450052, China; 2 Medical College of Henan University of Science and Technology, Luoyang 471003, China; 3 College of Basic Medical Sciences, Zhengzhou University, Zhengzhou 450001, China; 4 Henan Academy of Medical and Pharmaceutical Sciences, Zhengzhou University, Zhengzhou 450052, China; 5 Department of Clinical Laboratory, The First Affiliated Hospital of Zhengzhou University, No.1, Jianshe Road, Zhengzhou 450052, China

**Keywords:** Myricetin, 5-fluorouracil, Esophageal carcinoma, Chemosensitizer

## Abstract

**Background:**

Flavonoids are structurally heterogeneous, polyphenolic compounds present in high concentrations in fruits, vegetables, and other plant-derived foods. Currently, there is growing interest in the therapeutic applications of bioflavonoids for the treatment and prevention of diseases in humans. Myricetin is a naturally occurring flavonoid that is commonly found in tea, berries, fruits, vegetables, and medicinal herbs. Previous studies have shown that myricetin has antioxidant, anti-inflammatory and potent anticancer effects. It was interesting to investigate whether myricetin has the cooperative inhibitory effect combined with 5-fluorouracil on esophageal cancer cells.

**Methods:**

EC9706 cells were treated with 5-fluorouracil combination with or without myricetin. Colony formation assays, CCK-8 assay and flow cytometry were used to evaluate the chemosensitization activity of myricetin combine with 5-fluorouracil on the cell growth and viability, cell proliferation and apoptosis in vitro. Western blot was engaged to detect changes of Survivin, Cyclin D, Bcl-2, Caspase-3 and P53 protein expression level, which were associated with cells proliferation and apoptosis. Nude mouse tumor xenograft model was built to assessed chemosensitization effect of myricetin combine with 5-fluorouracil in vivo.

**Results:**

Compared with the 5-fluorouracil group without myricetin treatment, the groups treated with 5-fluorouracil combine with myricetin showed significantly suppressed cell survival fraction and proliferation, increased the cell apoptosis. Decreased Survivin, Cyclin D, Bcl-2, and increased Caspase-3, P53 expression level were aslo confirmed by western blot in 5-fluorouracil combine with myricetin groups in vitro. And in vivo assay, growth speed of tumor xenografts was significantly decreased in the mice treated with 5-fluorouracil + myricetin combiantion group.

**Conclusions:**

The study demonstrated both in vitro and in vivo evidence that combination of myricetin with 5-fluorouracil chemotherapy can enhance tumor chemosensitivity of esophageal cancer EC9706 cells, and myricetin could be a potential chemosensitizer for esophageal cancer therapy.

## Introduction

Esophageal cancer is a global health problem that ranked eighth in terms of incidence and sixth in terms of mortality [1],[2]. The northern regions in Henan province of China have the highest incidence of esophageal cancer, particularly the esophageal squamous cell carcinoma (ESCC) [3],[4]. Generally, the primary tumor of most patients can be cured with surgical resection, however, due to early distant metastases, the remainder of patients will eventually succumb to the disease. Systemic chemotherapy is regarded as one of the most effective treatments to improve survival [5]–[7]. However, the toxicity of most chemotherapy agents to normal tissues was the main obstacle to successful treatment. Therefore, to enhance efficacy and reduce toxicity become the tendency for chemotherapy regimens.

5-Fluorouracil (5-FU) was universally used as an anticancer agent in esophageal carcinoma [8],[9]. In order to improve the prognosis of patients with esophageal cancer, many previous studies were engaged to find more effective candidate mechanism to enhance 5-FU therapeutic effect with lower toxicity for normal tissues [10]–[13].

Most epidemiological evidence has shown that dietary habit and lifestyle play important roles in developing EC. And some studies indicated that tobacco and alcohol have roles in this cancer [14],[15], however, high consumption of vegetables and fruits could reduce the risk of EC [16],[17]. Flavonoids are structurally heterogeneous, polyphenolic compounds present in high concentrations in fruits, vegetables, and other plant-derived foods. Currently, there is growing interest in the therapeutic applications of bioflavonoids for the treatment and prevention of diseases in humans. Myricetin (3,3’,4’,5,5’,7- hexahydroxyflavone) is a naturally occurring flavonoid that is commonly found in tea, berries, fruits, vegetables, and medicinal herbs *et al.*[18],[19]. Previous studies have shown that myricetin has antioxidant, anti-inflammatory and potent anticancer effects [19]–[21]. It was interesting to investigate whether myricetin has the cooperative inhibitory effect combined with 5-fluorouracil on esophageal cancer cells.

In this study, we engaged esophageal squamous carcinoma cell line EC9706 to observe the effects of myricetin on combination with 5-fluorouracil enhances tumor chemosensitivity in vitro and in vivo, in order to provided a novel insight into myricetin as a potential agent for esophageal cancer chemosensitizers.

## Materials and methods

### Chemicals and cell culture

Myricetin and 5-FU (C_4_H_3_FN_2_O_2_) were purchased from Sigma-Aldrich, and dissolved in dimethylsulfoxide (DMSO) for storage at -20°C. Human esophageal squamous carcinoma cell line EC9706 was purchased from Shanghai Institutes for Biological Sciences, Chinese Academy of Sciences. The cells were routinely cultured in DMEM (Gibco, USA) supplemented with 10% heat-inactivated fetal bovine serum (FBS), 100 U/mL penicillin and 100 μg/mL streptomycin in a humidified cell incubator with an atmosphere of 5% CO_2_ at 37°C.

### Colony-forming survival assay

The overall survival of the cells treated with myricetin alone, 5-FU alone or in combination with different concentration, was assessed by the rate of colony formation. EC9706 cell were plated into 6-well plates and exposed to different concentration of myricetin (final concentration: 0 μM, 25 μM, 50 μM, 100 μM), After 4 hours later, 5-FU (10 μM, 20 μM, 40 μM, 80 μM, 160 μM) was added into the culture and maintained for 48 hours. 48 hours later, all cells were then washed, trypsinized, counted, and plated into 10 cm dishes containing DMEM supplemented with 10% FCS until colony formation was visible, which usually occurred in approximately 10-14 days. The colonies formed were stained with crystal violet, and the colonies with >50 cells scored as surviving colonies. The plating efficiency was calculated by dividing the average number of colonies per dish by the amount of cells plated. Survival fractions were calculated by normalization to the plating efficiency of appropriate control groups.

### In vitro cell proliferation assay

EC9706 cell lines in logarithmic phase growth were seeded into 96-well plates at a density of 1 × 10^4^ cells/well with three replicate wells. After 24 h incubation, when the cells were adhesive, the cells were exposed to a range of concentration of myricetin (0 μM, 25 μM, 50 μM, 100 μM), After 4 hours later, 5-FU (20 μM, 40 μM, 80 μM, 160 μM, 320 μM) was added into the culture. After exposed for 48 h, OD was measured by WST (water-soluble tetrazolium salt) assay using microplate computer software (Bio-Rad Laboratories, USA) according to the protocol of Cell Counting Kit-8 (CCK8) assay kit (Dojindo, Japan). 450 nm absorbance was read on a microplate reader (168–1000 Model 680, Bio-Rad, Hercules, USA). The curves of cell proliferation were plotted. Triplicate parallel experiments were performed for each concentration. The rate of inhibition was calculated by the following equation: rate of growth inhibition (%) = (OD_control_ – OD_treated_)/OD_control_ × 100%.

### Cell cycle analysis

Flow cytometry (BD FACS Canto II) was used to conduct cell cycle analysis. EC9706 cells treated with 0.1% DMSO (control) and different concentration of myricetin (combined with or without 5-FU) for 48 hours was trypsinized and washed twice with PBS. Then cell density was adjusted at 1 × 10^9^/L with 1 mL PBS and fixed in 70% cold ethanol overnight at 4°C. Adding 100 μL RNase A (1 g/L) into the cells, and keeping at 37°C for 30 min. Before the analysis, 400 μL of 10 mg/L propidium iodide (PI) was added into cells and then kept in dark for 30 min. The percentage of the cells in each phase of cell cycle was measured by FACScan system and the results were analyzed using CellQuest software.

### Cell apoptosis assay

Annexin V-FITC/PI apoptosis detection kit (Vazyme Biotech) was used to detect and quantify the presence of apoptotic cells. EC9706 cell lines in logarithmic phase growth were seeded in 6-well plate with 2.0 × 10^5^ cells in each well. Four groups (control group, 5-FU alone group, myricetin alone group, and combination group) cells were harvested and counted at 48 hours after incubated. The cell pellets were resuspended in 195 μL of binding buffer and stained with 5 μL each of annexin V-FITC and PI staining solution for 10 minutes at room temperature in the dark. Flow cytometry (BD FACS Canto II) was performed with the FACScan system using CellQuest software. Cell apoptosis rate was calculated as: (the number of cell apoptosis in each group/the total number of cells in each group) × 100%.

### Western blot analysis

Cell harvest was same as the method of cell apoptosis assay. Total protein was extracted from each group cells using RIPA buffer containing PMSF. A BCA protein assay kit (Beyotime, Haimen, China) was used to determine total protein concentration. Protein samples were resolved by electrophoresis of SDS-PAGE gels and transferred onto PVDF membranes. After blocking, the membranes were incubated overnight at 4°C with diluted primary antibody (the following antibodies were used: Survivin, Cyclin D1, Bcl-2, Caspase-3 and P53, 1:1000, Santa Cruz Biotechnology) and followed by incubation with an HRP-conjugated secondary antibody (1:1000, Santa Cruz Biotechnology). An antibody against GAPDH (Santa Cruz Biotechnology) served as an endogenous reference. Protein intensity were scanned on Typhoon PhosphorImager (GE Healthcare) for fluorescent signal. Experiments were performed in triplicate.

### Human tumor xenograft model in nude mouse

Immunodeficient female BALB/C nude mice, 5-6 weeks old, were from the Experimental Animal Center of Henan province, China. The mice were subcutaneously injected in the dorsal scapular region with EC9706 cells. In addition, a sufficient number of mice were implanted in order that tumors in a weight range as narrow as possible were selected for the trial on the day of treatment initiation (1 week after tumor cells implantation). The tumors were allowed to reach about 75-100 mm^3^ in size before the start of treatment. Then the mice were divided into four groups: myricetin group (25 mg/kg), 5-FU group (20 mg/kg), combination group, and saline control group. The mice were treated with chemotherapy using 3-week cycle regimen (×3 cycles) as previously described [22]. The tumor volume is measured with a caliper every 5 days, and tumor volume was calculated using the formula: volume = π (length × width^2^)/6. This study was complied with the NIH Guide for the Care and Use of Laboratory Animals. The protocol was approved by the committee on the Ethics of Animal Experiments of Zhengzhou University. All surgery was performed under sodium pentobarbital anesthesia, and all efforts were made to minimize suffering.

### Statistical analysis

SPSS 13.0 was used for statistical analysis. One-way analysis of variance (ANOVA) was used to analyze the significance between groups. Multiple comparisons were made using the Least Significant Difference test when the probability for ANOVA was statistically significant. All data represent mean ± SD. Statistical significance was set at *P* < 0.05.

## Results

### Myricetin increased chemosensitivity of 5-FU on tumor cells in vitro

**Figure 1:**
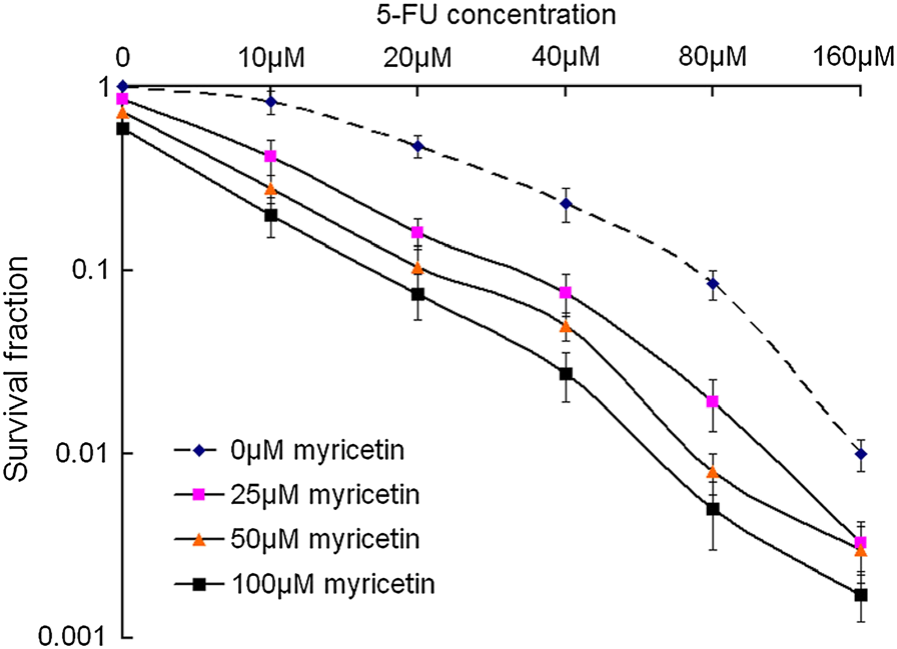
**Myricetin enhance chemosensitivity of 5-FU on cancer cells in vitro.** Cells were treated with indicated doses of myricetin and 5-FU for 48 h and colony formation assays were conducted. Chemosensitivity was measured by colony formation assay in the esophageal squamous carcinoma cell line EC9706. Compared to the control (0 μM myricetin), clonogenic survival of EC9706 cells treated with myricetin were decreased significantly under different 5-FU doses. Shown are averages of triplicate samples. Standard errors are shown by error bars.

To determine the effects of myricetin on tumor cells chemosensitivity, a clonogenic survival analysis was performed. It was found that treatment of the EC9706 cells with different concentration 5-FU alone could lead to inhibition effect on clonogenic survival. However, when combined with different concentration of myricetin, the surviving fraction decreased significantly (Figure 1).

### Myricetin enhances chemosensitivity on inhibiting the EC9706 cells proliferation

**Figure 2:**
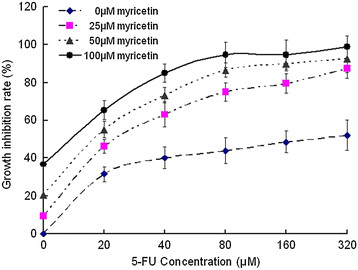
**Myricetin enhances 5-FU chemosensitivity on inhibiting the esophageal tumor cell proliferation.** Chemosensitivity was measured by CCK-8 assay in EC9706 cells treated with different concentration myricetin combine or not combine with 5-FU. Compared to the control group (0 μM myricetin), the proliferation inhibition rate of EC9706 cells were increased significantly in 5-FU chemotherapy combine with myricetin group.

CCK-8 assay was used to measure the effect of chemosensitization activity of myricetin on the growth and viability of EC9706 cells in vitro. Compared to the control (no myricetin and no 5-FU), the proliferation of EC9706 cells (Figure 2) was inhibited significantly in different consentration 5-FU group. It was also found that myricetin-only had but only limited inhibitory effect on tumor cells proliferation. However, a significant increase in tumor growth inhibition occurred for the cells treated with 5-FU combinated with myricetin. The result demonstrated that myricetin enhanced the chemosensitivity of EC9706 cells and has the function as a tumor chemosensitizer in esophageal squamous carcinoma cells in vitro.

### Myricetin combination with 5-FU enhances cell cycle arrest at the G_0_/G_1_ phase

**Figure 3:**
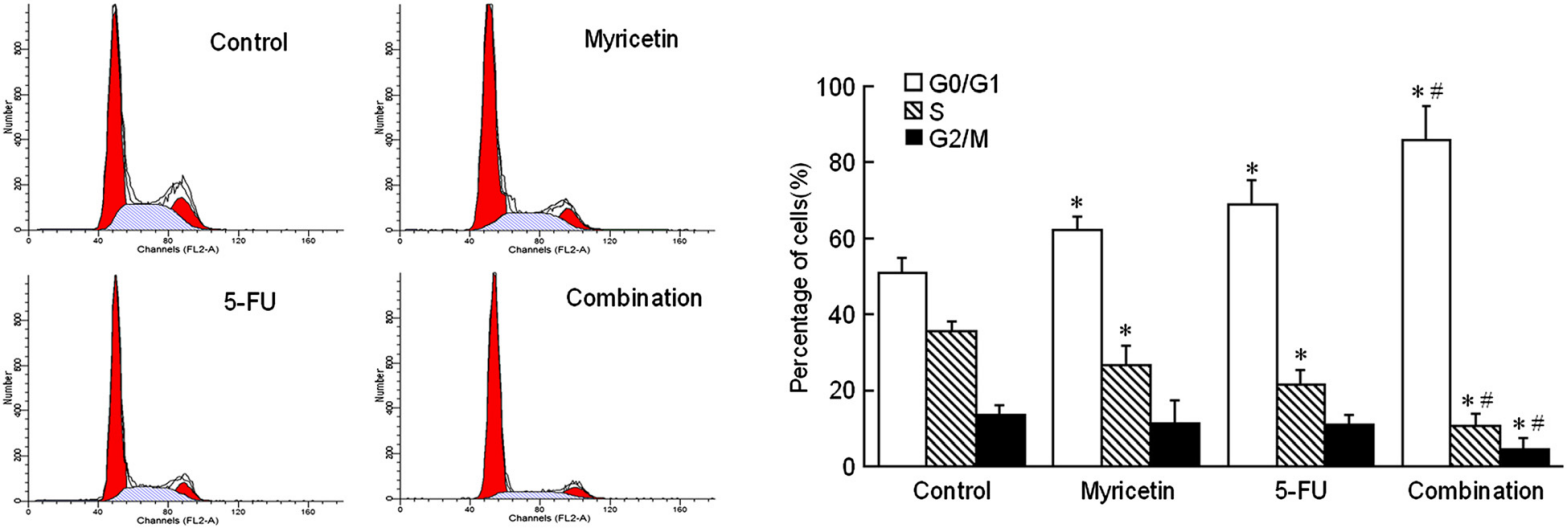
**Myricetin enhances 5-FU chemosensitivity on interrupting progression of cell cycle.** Cell cycle distribution was analyzed by flow cytometry. Data are presented as the mean of triplicate experiments. EC9706 cells incubated with 5-FU (80 μM) or myricetin (50 μM) showed increased G_0_/G_1_ phase percentage significantly, and decreased the S and G_2_/M phase fractions. And when 5-FU combination with myricetin, the effect of interrupting progression of cell cycle become more obvious (*P < 0.05 *vs* control, # *P* < 0.05 *vs* 5-FU group).

In order to examine whether the chemosensitivity of myricetin was related to cell cycle arrest, we next measured the cell cycle in EC9706 cells with flow cytometry analysis and PI staining. The phases distribution of cell cycle indicated that both 5-FU and myricetin could increase the percentage of EC9706 cells in G_0_/G_1_ phase and prevent cells entering into the S phase. As shown in Figure 3, EC9706 cells treated with myricetin or 5-FU resulted in a statistically significant increase in the G_0_/G_1_ phase that was accompanied by a decrease in the S phase. Furthermore when 5-FU combination with myricetin, the G_0_/G_1_ phase percentage increased significantly to 85.9%, compared with 5-FU alone (68.8%) or myricetin alone (62.1%). These results indicate that myricetin could enhance 5-FU chemosensitivity on cell cycle arresting in the G_0_/G_1_ phase, and delay the progression of the cell cycle, and inhibits proliferation of EC9706 cells.

### Myricetin enhances chemosensitivity of 5-FU on tumor cells apoptosis

**Figure 4:**
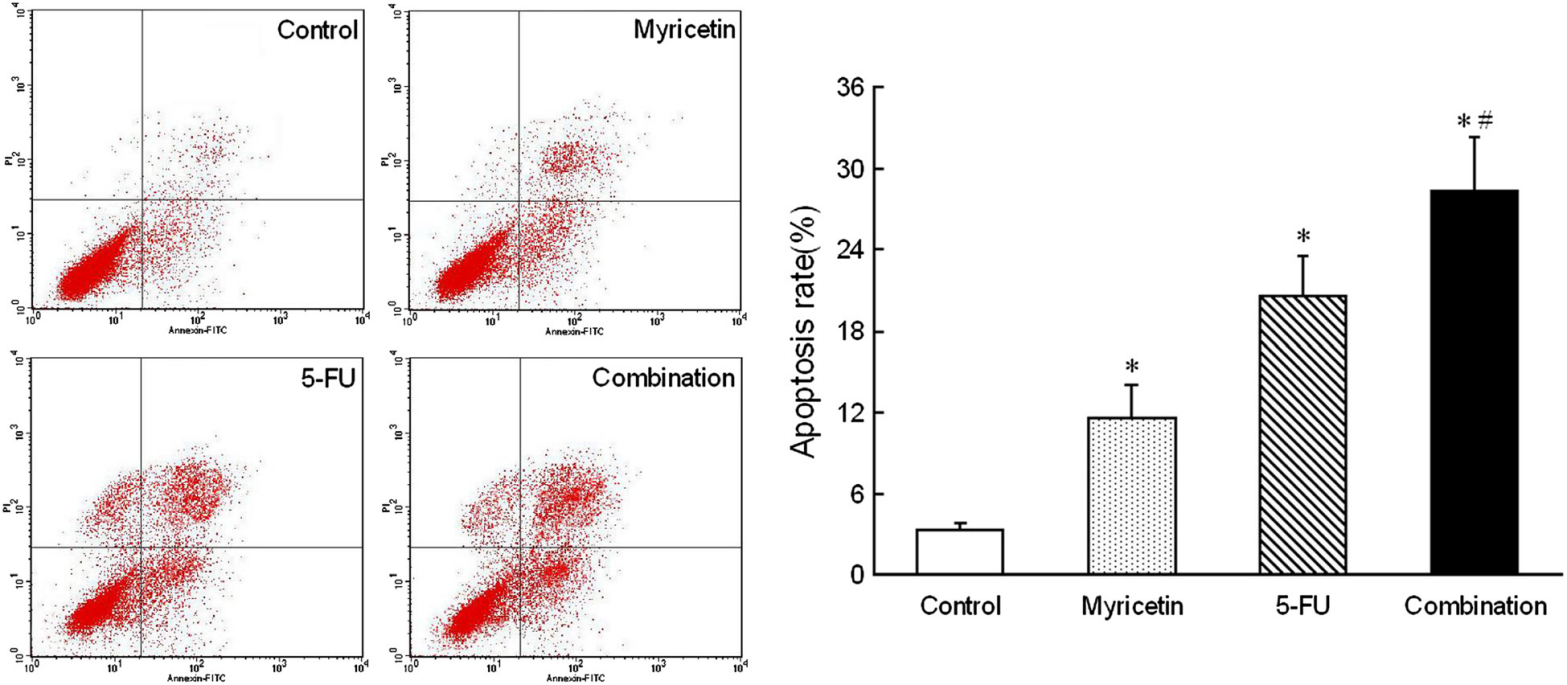
**Chemosensitizing effect of myricetin on tumor cells apoptosis.** Myricetin increase the chemosensitivity of 5-FU on apoptosis rate in EC9706 cells. The cells were stained by annexin V-FITC/PI, and cell apoptosis was analyzed by FCM. The data showed that apoptotic cells was statistically significant increased in 5-FU + myricetin combination group, compared to other three groups. (*P < 0.05 *vs* control, # *P* < 0.05 *vs* 5-FU group).

To investigate the apoptosis effect of myricetin-induced chemosensitization, we used flow cytometry (FCS) to measure the effect of myricetin and 5-FU on apoptosis in EC9706 cells. The cell stained with Annexin V-positive, PI-positive or both positive were defined to apoptosis cells. As shown in Figure 4. Compared to the control, both the apoptosis rate of EC9706 cells were increased in myricetin-alone group and 5-FU-alone group (*P* < 0.05). And furthermore, the apoptosis rate was significantly enhanced in 5-FU and myricetin combination group. Meanwhile, compared with the 5-FU alone group, the the apoptosis effect was obviously enhanced in 5-FU + myricetin combination group (*P* < 0.05).

### Myricetin enhances the 5-FU effection on alteration of the genes expression

**Figure 5:**
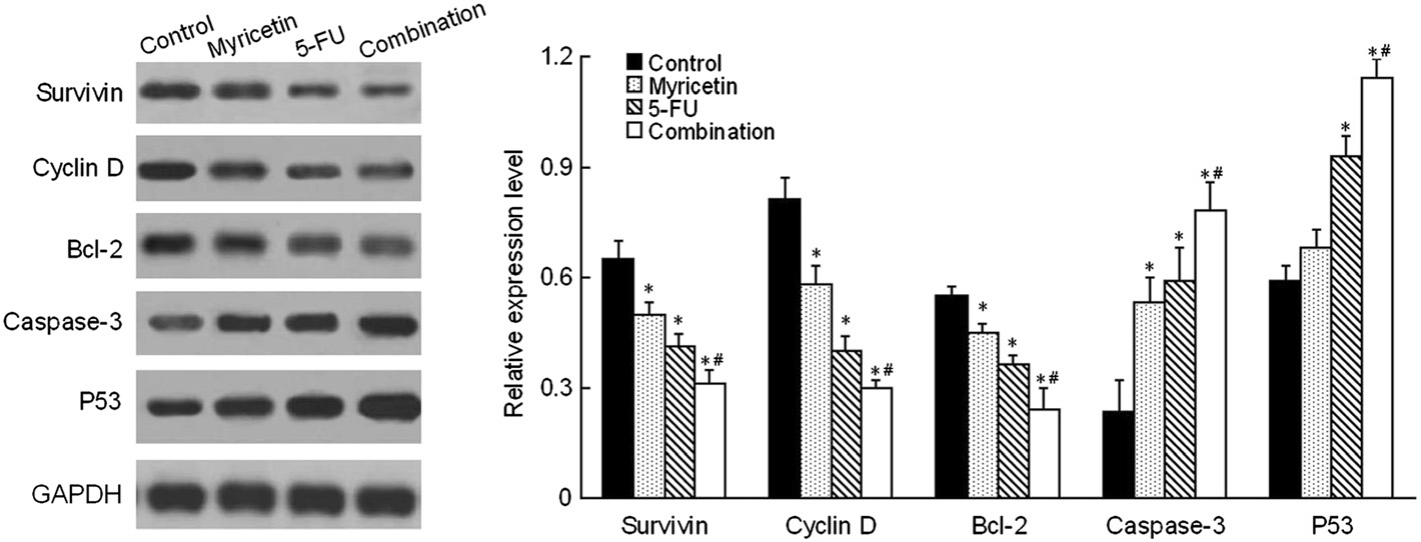
**Myricetin enhances the 5-FU effection on alteration of the genes expression.** EC9706 cells seeded in six-well plates were treated with myricetin (50 μM), 5-FU (80 μM) and myricetin +5-FU combination. After 48 h, total protein was extracted for western-blot. The relative expression level of Survivin, Cyclin D, Bcl-2, Caspas-3 and P53 from each independent experiments are shown. More alteration changes of these protein expression level were observed in myricetin and 5-FU combination group. Which indictated that myricetin in combination with 5-FU could promote the chemosensitivity of 5-FU in EC9706 cells by regulating the expression of Survivin, Cyclin D and Bcl-2, Caspase-3 and P53 (*P < 0.05 *vs* control, # *P* < 0.05 *vs* 5-FU). Data are presented as the mean of triplicate experiments.

After establishing myricetin enhanced 5-FU chemosensitivity to cell cycle arrest at G_0_/G_1_ and induced apoptosis, we sought to investigate the underlying molecular mechanism. To examine whether myricetin in combination with 5-FU promotes the chemosensitivty of 5-FU in EC9706 cells by regulating the expression of Survivin, Cyclin D1, Bcl-2, Caspase-3 and P53 protein, we engaged western-blot to checked these protein expression levels (Figure 5). Compared to the control, the expression levels of Survivin, Cyclin D1 and Bcl-2 in both myricetin and 5-FU group were decreased, while the Caspase-3 and P53 level was increased (*P* < 0.05). Furthermore, more alteration changes of these protein expression level were observed in myricetin and 5-FU combination group. The expression level of Survivin, Cyclin D1 and Bcl-2 in myricetin and 5-FU combination group were more lower than in myricetin alone or 5-FU alone group (*P* < 0.05), while more higher expression level of Caspase-3 and P53 in combination group than in myricetin alone or 5-FU alone group (*P* < 0.05). These clear results explained that myricetin in combination with 5-FU could promote the chemosensitivity of 5-FU in EC9706 cells by regulating the expression of Survivin, Cyclin D1, Bcl-2, Caspase-3 and P53, when compared with 5-FU treatment.

### The chemosensitizing effect of myricetin in vivo

**Figure 6:**
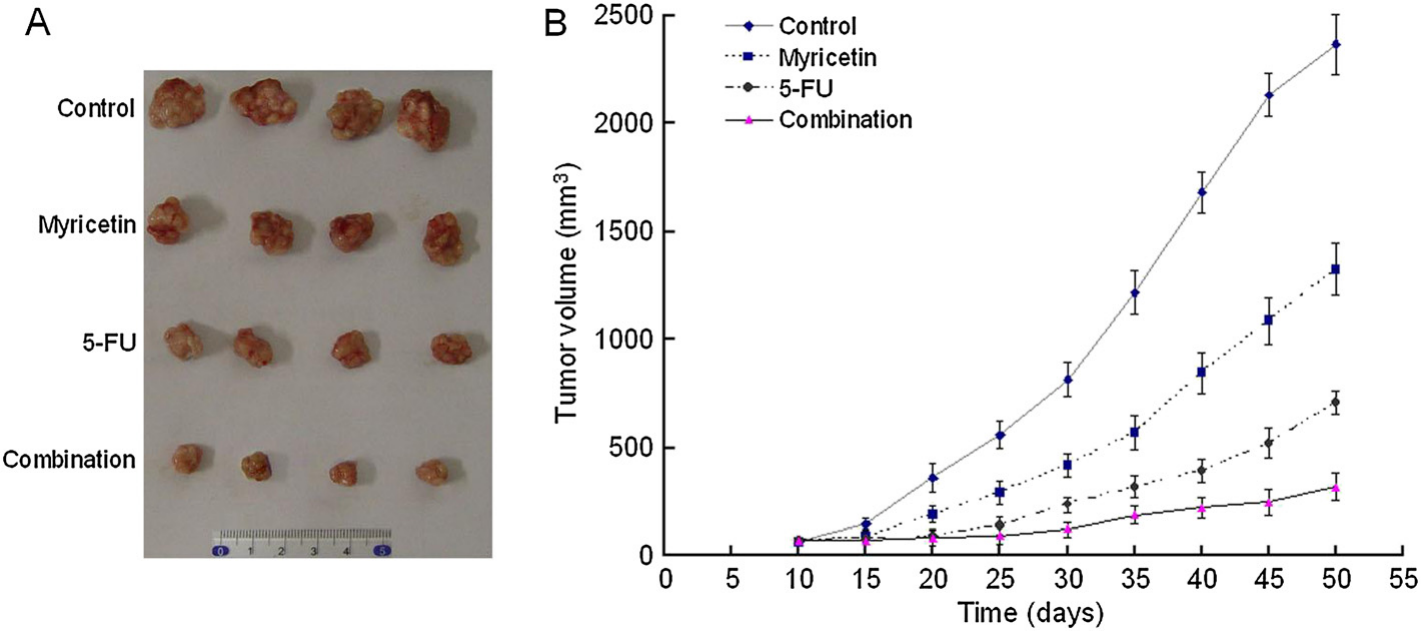
**EC9706 cell xenograft tumor for detecting myricetin sensitizing tumors to 5-FU treatment in vivo.** EC9706 cells were implanted subcutaneously in immunodeficient female BALB/C nude mice. Mice treated with myricetin alone, 5-FU alone, and myricetin + 5-FU combination. **A**. The xenograft tumors from nude mice in each groups after 2 months implantation. **B**. Shown are medium tumor weights of each group as function of time after implantation. The data curve showed that 5-FU combination with myricetin could inhibit the tumor growth significantly, compared with other three groups.

Because myricetin has the chemosensitization activity on cell survival, proliferation and apoptosis in vitro, we performed a proof-of-principle experiment using a esophageal cancer EC9706 cell xenograft mice model to determine chemosensitizing effect of myricetin in vivo. We found that myricetin-alone group had less inhibitory effect on tumor growth, compared with tumors treated with 5-FU alone (Figure 6). However, a significant slower down in tumor growth occurred for mice treated with 5-FU combination with myricetin. These results demonstrated that myricetin could increase esophageal tumor cell killed by 5-FU in vivo, and further indicated that myricetin can function as a powerful chemosensitizer for esophageal cancer.

## Discussion

Esophageal carcinoma is one of the most aggressive malignancies and has become an emerging public health problem for its high morbidity and mortality [23],[24]. More effective treatments, as well as methods for earlier diagnosis, have led to improved survival over recent decades. However, patients with esophageal cancer still exhibit rapid progression and poor prognosis, owing to extensive local invasion, lymph node involvement and distant metastases at the time of diagnosis [25],[26]. Consequently, more effective chemotherapies have become an important means of extending the survival of esophageal cancer patients. 5-FU, a pyrimidine analogue of road-spectrum anticancer drug, is widely used in chemotherapeutic regimens [27], but the large side effects which it has been shown are a still limit aspect for use. The development of novel strategies for inhibiting tumor cells growth and enhancing chemosensitivity are the focus of much medical research.

Over the last few years, interest in exploring the use of traditional medicines for the prevention or treatment of tumors has increased. Myricetin is a naturally occurring flavonol and commonly ingested through our diet in many natural foods and medicinal herbs. There are studies showed that myricetin has the function to inhibit cellular proliferation and to induce apoptosis in tumor cells [28],[29]. Acquired data have indicated the biological activities of myricetin, such as anti-oxidant, -inflammatory, -carcinogen, -viral, and apoptosis induction, which could protect against tumorigenicity. Therefore it was reasonable to hypothesize that myricetin might function as a chemosensitizer.

To test this hypothesis, we firstly conducted colony formation assays and prolifetation assay in esophageal cancer cell line (EC9706), It was found that treatment of the cells with 5-FU chemotherapy combined with myricetin could significantly decrease the survival fractions and prolifetation of cancer cells. And myricetin exhibited obvious effect of enhancing chemosensitivity with 5-FU on the EC9706 cell survival and prolifetation. In addition, the growth and proliferation of cancer cells are mediated via cell cycle progression [30]. The inhibition of the cell cycle has become an appreciated target for management of cancer [31]. In this study, we also examine the chemosensitivity of myricetin and 5-FU on cell cycle. Which indicated that myricetin could enhance the 5-FU inhibition effect on the proliferation of cancer cells by arresting the G_0_/G_1_ phase. Meanwhile, Previous studies had established that Cyclin D1 plays a crucial role in the progression of cell cycle from G_1_ to S phase and the down-regulation of Cyclin D1 would lead to cell cycle arrest at G1 [32]. In present study, we found that Cyclin D1 were downregulated in myricetin and 5-FU combination treated group, and Cyclin D1 expression level was lower than in myricetin alone and 5-FU alone groups. These results indicate that myricetin could enhance 5-FU chemosensitivity on cell cycle arresting, and delay the progression of the cell cycle, and inhibits proliferation of EC9706 cells.

On the other hand, Apoptosis was thought to be the major reason of cell death induced by chemosensitizer. It is now widely recognized that drug-induced apoptosis may be used to measure the sensitivity of cells to drugs, with an increased rate of apoptosis meaning that the cells have a higher sensitivity to chemotherapy [33],[34]. We examined the apoptotic effect induced by myricetin combined with 5-FU or administered individually. The results indicated that myricetin and 5-FU alone significantly induced apoptosis compared with the control, and that the combined treatment effects were stronger than the individual effects of myricetin and 5-FU. And to further test the chemosensitizing mechanism of myricetin on tumor cells apoptosis, we detected the expression level of Survivin, Bcl-2, Caspase-3 and P53, which play very important role during cells apoptosis [35]– [40]. The data showed that 5-FU combine with myricetin could be more efficient to decrease Survivin, Bcl-2 expression, and increase the Caspase-3, P53 expression, which aslo indicated that myricetin could enhanced the 5-FU chemosensitivity of apoptosis.

And in this study, we also engaged esophageal tumor bearing nude mice model to observe the chemosensitizing effect of myricetin in vivo. The result showed the tumor growth speed was significantly slower down in occurred for mice treated by 5-FU combine with myricetin. These data above demonstrate that myricetin has a chemosensitization potential in vitro and in vivo, and further indicated that myricetin can function as a powerful chemosensitizer for esophageal cancer treatment by 5-FU.

In conclusion, the results of the current study demonstrate the effect of myricetin on combination with 5-FU enhances esophageal tumor chemosensitivity in vitro and in vivo, it could provided a novel insight into myricetin as a safe and potential agent for esophageal carcinoma chemosensitizers to enhance the effectiveness of chemotherapy.

## Competing interests

The authors declare that they have no competing interests.

## Authors’ contributions

LW, SJZ and GQZ designed the study, LW, JFF, XNC, WG and YWD carried out the experiments and drafted the manuscript; YYW, WQZ, SJZ and GQZ participated in the experiments and data analysis. All of the authors approved the final version of the manuscript.
